# The Way to Modern Shutter Speed Measurement Methods: A Historical Overview

**DOI:** 10.3390/s22051871

**Published:** 2022-02-27

**Authors:** Gyula Simon, Gergely Vakulya, Márk Rátosi

**Affiliations:** 1 Alba Regia Technical Faculty, Óbuda University, 8000 Székesfehérvár, Hungary; vakulya.gergely@amk.uni-obuda.hu; 2 Department of Computer Science and Systems Technology, University of Pannonia, 8200 Veszprém, Hungary; ratosi@dcs.uni-pannon.hu

**Keywords:** exposure time, shutter speed, direct method, motion blur, equivalent sampling

## Abstract

Exposure time is a fundamental parameter for the photographer when the photo is composed, and the exact length of the exposure may be an essential determinant of performance in certain camera-based applications, e.g., optical camera communication (OCC) systems. There can be several reasons to measure the shutter speed of a camera: shutter speed may be checked at the time of manufacturing; it may be necessary to recheck in case of an elder camera model; it may be necessary to be measured if its exact value is not provided by the manufacturer; or a precise measurement may be necessary for a demanding application. In this paper various methods for shutter speed measurement are reviewed, presenting and analyzing methods that are still relevant today either for manufacturers, service personnel, amateur photographers, or the developers of camera-based systems. Each presented method is illustrated by real measurement results and the performance properties of the methods are also presented.

## 1. Introduction

Exposure time (often referred to as shutter speed) is the length of time for which the film of a traditional camera or the sensor of a digital camera is exposed to the incoming light in order to create the image. In most cases, darker scenes (e.g., night time photos) require longer exposure times, while bright items (e.g., a sunny landscape) can be photographed with short exposure times. The exposure time has crucial effect in photography when a picture is composed: short exposure times allow for catching fast movements, while long exposure times allow motion blur and thus create artistic effects [1]. These effects are so important parts of the photographic experience that even virtual reality photography simulates them [2]. While the special or individual choice of exposure time (and the corresponding aperture) allows for expressing the photographer’s creativity and feelings about the scene, technical systems may use various exposure times to create optimal results, e.g., in High Dynamic Range (HDR) photography [3,4,5,6]. Imaging systems are utilized in various technical fields, where the shutter speed is set to achieve the requirements of the application; e.g., when high speed fluid flows are measured using interferometry, the exposure time is set very short (as low as a few microseconds) to prevent motion blur [7,8], while in astronomical photography extremely long exposures times (even days) may be used to provide good signal to noise ratio [9,10,11]. Object tracking systems usually require short exposure times to provide sharp images [12,13,14]. For speed estimation, longer exposure times may also be utilized, where the amount of motion blur contains information about the speed of the object [15,16,17]. The ubiquity of smartphones, equipped with good quality cameras, stimulates the research of OCC systems. Here, the cameras are utilized as sensors, to receive visually coded information [18,19,20], and the value of the exposure time (among other factors) determines the data rate, the bit-error-rate, and the achievable communication distance [21,22,23]. In camera-based positioning systems, OCC is often utilized to allow beacon identification [24,25].

**Figure 1:**
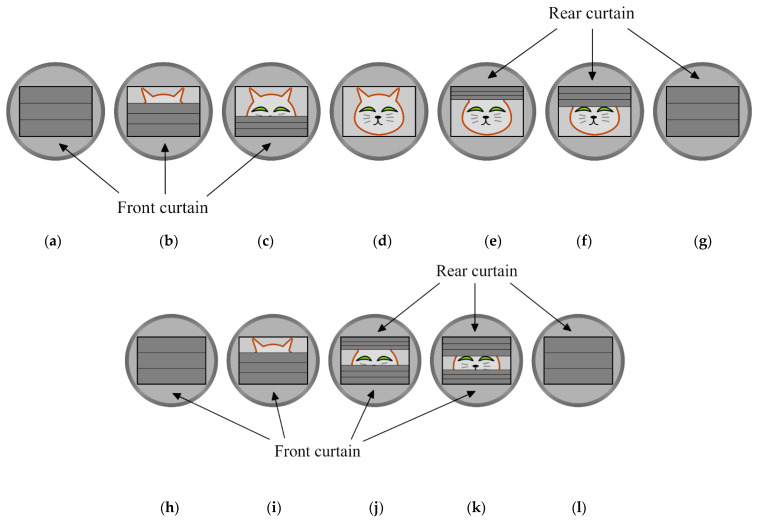
The operation of a mechanical focal plane shutter with two curtains. (**a**–**g**) long exposure time, the shutter is fully open, (**h**–**l**) short exposure time, the only a band in the shutter is open.

In most cameras, which use mechanical shutters, the exposure time is determined by two moving shutter curtains, deployed in front of the focal plane. The mechanism, called rolling shutter, is illustrated in Figure 1. The upper and lower rows illustrate the cases of longer and shorter exposure times, respectively. Before the exposure, the first (or front) curtain blocks the way of light, and thus the shutter is closed, as shown in Figure 1a. At the beginning of the exposure, the front curtain starts to fall, opening the shutter so light can reach the sensor (see Figure 1b,c). For longer exposure times, the shutter may be fully open for a while, as shown in Figure 1d. After a while, the second (rear) curtain starts to roll down (Figure 1e,f) and finally it closes the shutter again, as shown in Figure 1g. For shorter exposure times, the process starts similarly, as shown in Figure 1h,i, but the shutter may not be completely open: the rear curtain starts to fall while the front curtain is still falling (see Figure 1j). In this case, a part of the picture is covered by the front curtain, and another is by the rear curtain. Both curtains having the same speed, the narrow gap between the curtains rolls down, as shown in Figure 1j,k. It is clear from the operation that the exposure is not abrupt: first the upper part of the sensor (or film) is exposed to the light, and then gradually, as the front curtain falls, the lower part is also exposed. The closure is similar: starting at the upper part, the sensor is gradually blocked from light as the rear curtain falls. 

Nowadays, most of the smaller sized and inexpensive digital cameras use electronic shutters, which require no mechanical parts. In this case, the light sensor is switched on and off for the time of the exposure. Electronic shutters may behave very similarly to their mechanical rolling shutter counterparts: for easier operation, the sensor is operated line-wise: first, the uppermost line of pixels is switched on (exposed and then the sensor values are stored), then the second line follows, and so on to the last line. Thus, the upper part of the picture is exposed somewhat earlier than the lower part, similar to the case of Figure 1, causing distortion in the case of a moving target. More expensive digital cameras, however, may contain global shutter mechanisms, where every part of the sensor is switched on and off (and thus exposed) at the same time. Such cameras may provide distortion-free pictures for demanding industrial or scientific applications.

Technically, the exposure time is defined as the time span for which the center of the sensor is exposed to the incoming light. This definition is valid for both rolling shutter and global shutter cameras: although in rolling shutter cameras various parts of the image are exposed at different times, every part has (approximately) the same exposure time. Global shutter cameras simply expose every part of the picture at the same time for the time span of the exposure time.

The exposure time of a camera can usually be set in discrete steps, e.g., by the exposure dial on a traditional camera or by software in case of a digital camera. The exact value of the exposure time, however, may differ from the nominal value: old mechanical shutters may deteriorate in time and the shutter speed may significantly differ from the nominal values, and the actual exposure time of a digital camera may also differ from the nominal value reported by the manufacturer. It also happens that manufacturers of inexpensive cameras do not provide timing values at all. Thus, the measurement of the exposure time may not be performed only during manufacturing and production control by the manufacturer itself, but the critical user may also need to measure it if the camera is used for high precision applications.

Various camera types and different accuracy requirements led to the development of several methods to measure the exposure time of cameras, the earliest solutions dating back to the 1890s, when electro-opto-mechanical equipment were proposed to measure the Speed of Camera Shutters [26]. In this paper, those measurement principles and approaches are revised, which still has some relevance today, also showing a historical path towards modern solutions. In addition to the introduction of the measurement methods, their performance properties (accuracy, measurement range) will also be discussed and illustrated. The following type of methods will be discussed in detail:

- The direct method allows the measurement of shutter speed by observing the operation of the mechanical shutter mechanism. This method requires access to the camera’s focal plane: for vintage and traditional film cameras it is straightforward, but for most digital cameras it is only possible during the manufacturing process;
- The most common indirect way to measure the shutter speed is taking photos of a moving object and calculate the exposure time from the motion blur, observed on the picture, and the speed of the moving object. For simple measurements, the moving object can be a real physical object with known velocity, but more precise measurements use electronically simulated movements;
- The shutter time of cameras capable of recording video streams can be measured using equivalent sampling. In this case, a blinking light source is recorded by the camera under test, and from the change of the recorded light intensity vs. time, the shutter speed is calculated.

This paper is organized as follows: in Section 2, the test equipment, used for illustration throughout the paper, is introduced. In Section 3, the direct method is presented. In Section 4 various methods, based on motion blur, are discussed. In Section 5, a different approach is presented, which uses equivalent sampling of a blinking light source. Each method is illustrated by real measurements and their performance properties are evaluated. In Section 6, the discussed methods are compared.

## 2. Test Equipment

**Figure 2:**
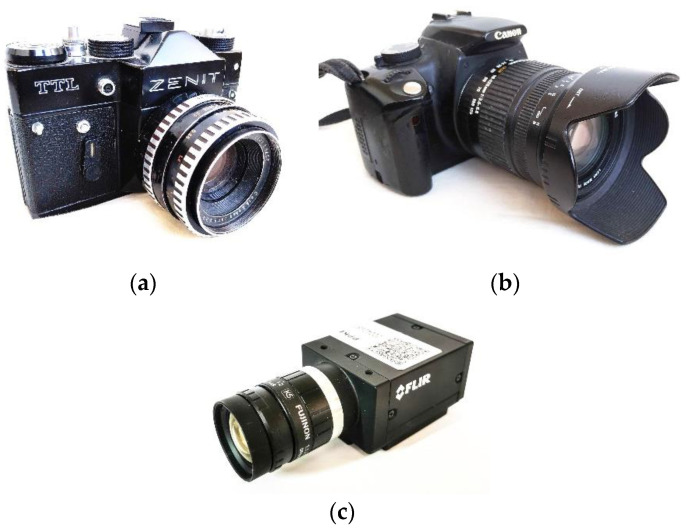
Cameras used for testing. (**a**) Zenit TTL (C1), (**b**) EOS 350D (C2), (**c**) Grasshopper3 (C3).

Three cameras from different eras were used to illustrate the measurement processes and their performance properties. The cameras are shown in Figure 2.

The oldest model is a Zenit TTL SLR (Single Lens Reflex) film camera from the late 70 s, produced by KMZ (Krasnogorsk, Soviet Union), shown in Figure 2a. It has an all-mechanical cloth shutter with 5 selectable exposure times from 1/30 s to 1/500 s. From now on, this camera will be referred to as C1.

The EOS 350D, shown in Figure 2b, is one of the earliest DSLR (Digital Single Lens Reflex) cameras produced by Canon (Tokyo, Japan). It is equipped with an electromechanical shutter system with exposure times between 1/4000 s and 30 s with 1/3 f increments. This camera will be referred to as C2.

The third camera, C3, is the FLIR Grasshopper3 (GS3-U3-23S6M), which is an industrial camera mainly targeted for machine vision purposes, produced by Teledyne FLIR (Wilsonville, OR, U.S.). It is equipped with a global electronic shutter and can take photos and videos with predefined exposure times from 8 μs to 31.9 s. The camera is shown in Figure 2c.

The properties of the cameras, relevant to this research, are summarized in Table 1.

## 3. Direct Method

A wide range of direct methods has been applied to measure exposure time, the common factor between them being that a light source is used in front of the shutter and the length of the passing light pulse is measured behind the shutter. The earliest systems used the film itself as a sensor [26], but later electronic sensors (e.g., photocells, photodiodes, phototransistors) were utilized [27,28]. The length of exposure was often estimated by using a series of light sources [29] or stroboscopic effect [26]; thus, the time measurement could be replaced by counting. Other solutions integrated the sensed light pulse by a capacitor and calculated the length of the pulse from it [27]. Later devices used digital circuits to present the measurement results for the user [28]. Using the same measurement principle, smartphone apps with simple external hardware, to be connected to the microphone input, are available for modest accuracy requirements [30]. Professional equipment using direct method can measure the shutter time with 5–10 μs uncertainty [31,32].

**Figure 3:**
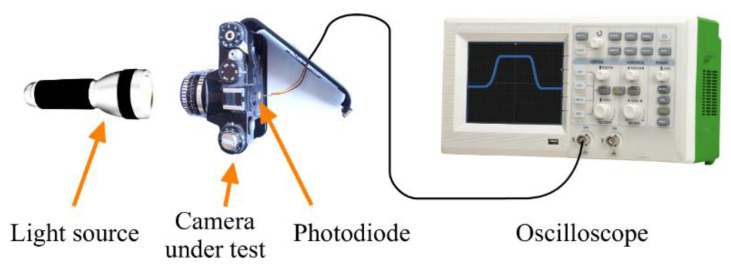
Direct method using a light sensor and an oscilloscope.

ISO standard 516 also defines a direct measurement method to determine the shutter speed of a camera [33]. The measurement scheme is shown in Figure 3. A constant illumination is provided in front of the camera, which has a light sensor (e.g., photodiode or phototransistor) placed behind its shutter at the center of the focal plane. The detected light intensity is observed on an oscilloscope. During the exposure, the light sensor detects increased light intensity. Thus, the width of the detected impulse is the exposure time.

**Figure 4:**
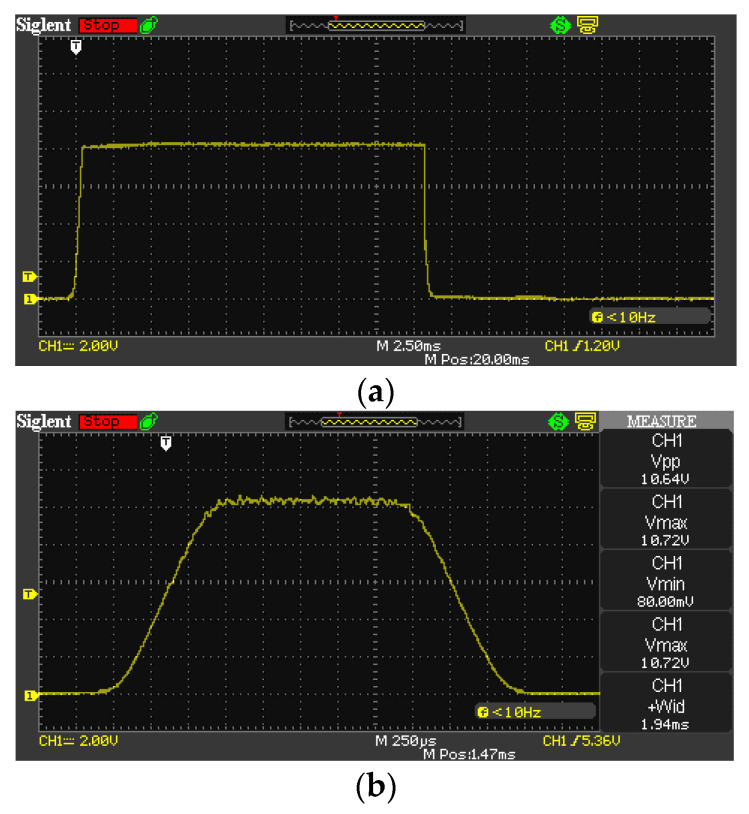
Measurements of C1 using the direct method. Settings: (**a**) 1/30 s, (**b**) 1/500 s.

A set of measurements are shown in Figure 4. The first measurement was made with exposure time 1/30 s, but the measurement shows significantly different time of 23.4 ms = 1/43 s. The second measurement was made with setting 1/500 s, and the corresponding measurement shows 1.94 ms = 1/515 s.

**Figure 5:**
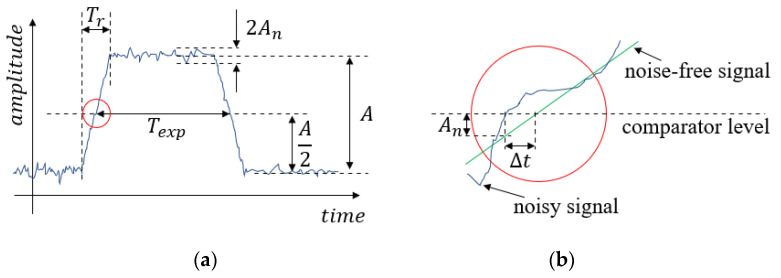
Measurement error of the direct method due to measurement noise. (**a**) Measured quantities, (**b**) measurement error of the rising edge.

In the direct method, the exposure time is read directly from the oscilloscope. If the pulse width is determined from the screen of the oscilloscope by the user, the reading uncertainty $h_{read}$ can be as high as 2–5%. However, digital oscilloscopes provide built-in measurement features, typically reducing the reading error to approx. 0.5%. In addition to the reading error, measurement noise may cause uncertainty in the measurement, as follows. Let us suppose that the magnitude of the noise is $A_{n}$, the measured signal amplitude is A, and the rising and falling times of the signal are $T_{r}$, as shown in Figure 5.

If the slope of the signal is m then the time measurement uncertainty Δt, caused by the magnitude uncertainty $A_{n}$, is

(1) $$\Delta t=\frac{A_{n}}{m}=\frac{A_{n}}{A/T_{r}}=T_{r}\frac{A_{n}}{A}=\frac{T_{r}}{SNR},$$

where $SNR=A/A_{n}$ is the signal-to-noise ratio. Since the uncertainty is present on both the rising and falling edges, in the worst case the measurement uncertainty $\Delta T_{exp}$ will be

(2) $$\Delta T_{exp}=2\Delta t=\frac{2T_{r}}{SNR}.$$

Since $T_{r}$ can be considered constant (it is the time while the curtain moves the distance equivalent to the light sensor’s size), the measurement uncertainty depends only on the signal-to-noise ratio. The relative uncertainty $h_{exp}$ of the exposure time measurement is the following:

(3) $$h_{exp}=\frac{2}{SNR}\frac{T_{r}}{T_{exp}}.$$

**Figure 6:**
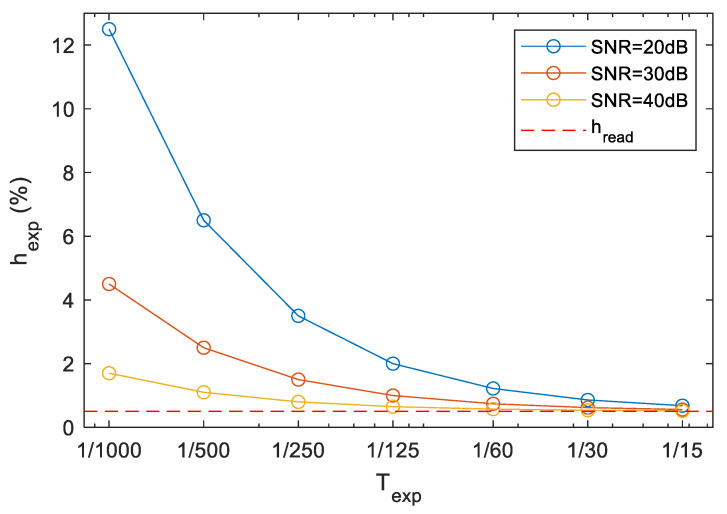
Relative measurement uncertainty of the direct method.

According to (3), the relative measurement uncertainty, due to noise, increases for small exposure times. The total relative uncertainty, as the sum of the reading error $h_{read}$ and noise uncertainty $h_{exp}$, is shown in Figure 6, for the measured camera C1. The value $T_{r}$ was set to 0.6 ms, as was measured in case of C1 (see Figure 4b). The *SNR* in the measurements was close to 20 dB, thus the blue curve shows the maximum expected relative uncertainty for camera C1. For the estimation of (3), the values A and $T_{r}$ were simply read from the scope, and $A_{n}$ was estimated as the RMS of the horizontal section of the measured signal.

The measurement results of camera C1 are shown in Table 2. The results show that the camera has a large bias at the longer exposure time region, the largest error is almost 30% at 1/30. These errors are much higher than the expected maximum measurement error, shown in Figure 6; thus, the camera surely has problems with its timing. Considering the age of the camera, it is not surprising. At shorter exposure times, the camera shows acceptable performance.

Comparing the results of Table 2 with the measurement errors in Figure 6, the following statements can be made:

- The camera behaves reasonably well at shutter speeds of 1/500 and 1/250, the error is around or below 3–4%. In this range, the uncertainty of the measurement is comparable to the error of the camera, thus the error level cannot be determined more precisely.
- At longer exposure times, the error of the camera is much higher. Since here the uncertainty of the measurement is smaller, it can be stated that the shutter time error of the camera at 1/30, 1/60, and 1/125 is 30 ± 1%, 14 ± 2%, and 8 ± 3%, respectively.

The direct method proposed in the standard can be used for a wide range of cameras, but unfortunately access to the focal plane is necessary to perform the measurement. In traditional film cameras, it can be done easily by opening the back cover and placing the sensor in place of the film [34]. In most digital cameras, however, access to the focal plane is not possible without breaking the integrity of the camera; thus, the application of the direct method is limited to the manufacturing phase or service activities; regular users must use other indirect and non-intrusive methods, which utilize pictures taken in the normal operation mode of the camera.

Notice that the exposure time is defined at the center of the image. Although there might be slight differences in the exposure times in different parts of the picture, depending on the actual properties of the shutter, this variation is usually neglected when indirect methods are utilized.

## 4. Methods Based on Motion Blur

Since cameras integrate the incoming light during the interval of the exposure time, the image taken on a moving object may be blurred. The blurring effect depends on the speed of the object (the higher the speed the higher the blur) and the exposure time (the longer the exposure time the higher the blur). The latter effect can be utilized to measure the length of the exposure.

The method was applied in many forms to provide an estimate on the exposure time. Since rotating movements are easier to handle in measuring equipment than lateral movements, numerous methods used some form of rotating target; e.g., in the first published method a rotating disk with holes was applied [26], or later a camera was rotated while taking a photo on a small fixed light source [35]. For the sake of convenience, in simple measurement setups often conventional turntables were used to create controlled movement, as will be described in Section 4.1. Later, instead of moving physical objects, electronic systems were utilized to simulate movement. In the era of cathode ray tubes (CRTs), the swiping electron beam on the display provided the moving target, which allowed higher precision and wider measurement range, as will be shown in Section 4.2. Today’s measurement equipment utilizes *LED* arrays, which will be discussed in Section 4.3.

### 4.1. Moving Phisycal Target

**Figure 7:**
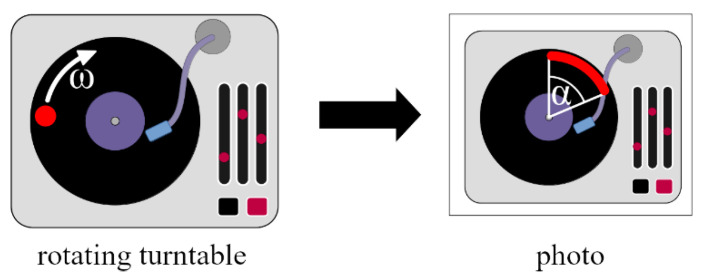
Motion blur method with a rotating object on a turntable.

In a convenient measurement setup, an image is taken of a small light object, which is rotated with known angular velocity. During the time of the exposure, the object will move, and so on the picture blurring will occur; instead of a point, an arc will be shown. For the sake of convenience, a turntable may be used for driving purposes [36], and the measurement process may be automated [37]. In Figure 7, a measurement setup with a turntable is shown. The angular velocity ω of the turntable is known, e.g., $\omega =33\frac{1}{3}$ RPM (rotations per minute) or 45 RPM, and the measured angle of the blur is α. The length of the exposure $T_{exp}$ can be calculated as follows:

(4) $$T_{exp}=\frac{\alpha }{\omega }.$$

**Figure 8:**
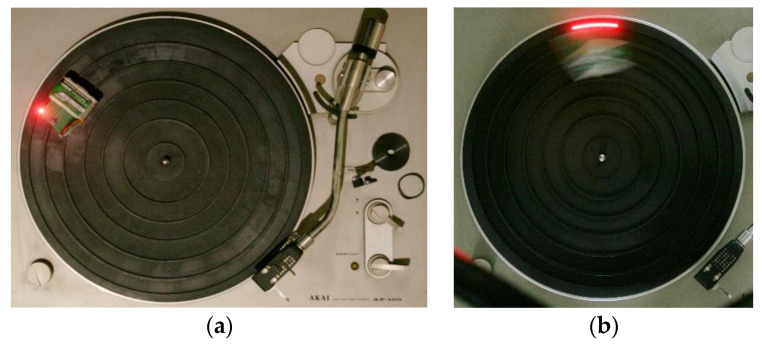
(**a**) The turntable with an *LED* light source. (**b**) Measurement with $T_{exp}=1/\;10$ s.

The measurement equipment and a photo taken with $T_{exp}=1/10$ s can be seen in Figure 8.

The measurement uncertainty of (4) depends on the accuracy of angular velocity ω and the accuracy of measurement α. Since normally the uncertainty of ω is negligible compared to that of α, the uncertainty Δt can simply be approximated as follows:

(5) $$\Delta T_{exp}=\frac{\Delta \alpha }{\omega },$$

and the corresponding relative uncertainty is

(6) $$h_{exp}=\frac{\Delta T_{exp}}{T_{exp}}=\frac{\Delta \alpha }{\alpha }.$$

**Figure 9:**
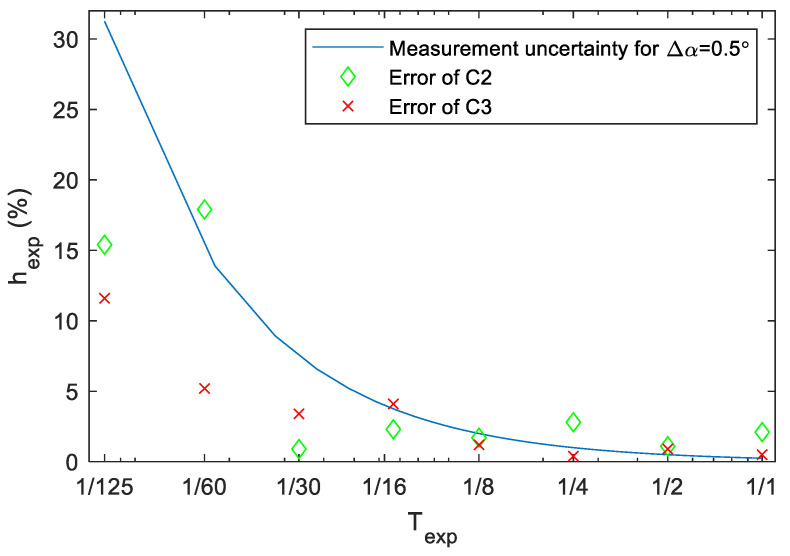
Relative measurement uncertainty of the turntable measurement.

Since the maximum reading uncertainty of angle α on a photo is approx. Δα=0.5 degrees, based on our experiments, and the uncertainty is independent of the actual value of α, the relative uncertainty (6) is practically inversely proportional with α. The maximum relative uncertainty of (6) are shown in Figure 9 for a turntable with $\omega =33\frac{1}{3}$ RPM, where the measured angle (in degrees), as a function of exposure time is the following:

(7) $$\alpha =T_{exp}\cdot 360{}^{\circ}\cdot \frac{33\frac{1}{3}}{60\;\sec}=200{}^{\circ}/\sec\cdot T_{exp}$$

According to the results shown in Figure 9, the uncertainty is around 1%, when the measured exposure time is higher than 1/4 s. For exposure times shorter than 1/40 s, the relative measurement uncertainty may be higher than 10%; thus, this measurement method is suitable only for long exposure times.

Measurements for cameras C2 and C3 were made by the turntable method, the results are shown in Table 3, and the measured relative errors are also plotted on Figure 9. In the case of C3, the error trend is similar to the theoretical measurement uncertainty, although the error is somewhat lower at shorter times, indicating a smaller reading uncertainty than 0.5 degrees. Since the error in this case is lower than the measurement uncertainty, it can be stated that the camera is more accurate than the measurement itself. In the case of C2, however, the error is significantly higher at longer exposure times: here, the camera has detectable deviation (in the range of 1–2%) from the nominal exposure values.

### 4.2. Moving Electron Beam: CRT Monitor

**Figure 10:**
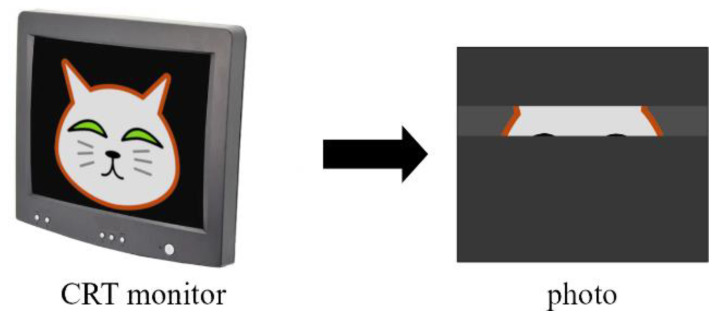
Measurements using a CRT monitor.

Instead of mechanical movement, a moving electron beam can be used to estimate the exposure time [36]. A few decades ago, Cathode Ray Tubes (CRTs) were generally used in monitors and TV sets. This equipment provided an easily available and straightforward way to produce the necessary moving electron beams for the measurement. The principle is shown in Figure 10. The CRT display creates the picture row-by-row, moving the electron beam from left to right in each row, then starting at the beginning of the next row. The picture is drawn on the screen frequently enough (60–120 times per second) so that the human eye does not see the blinking. When a photo is taken of the display, only those rows are shown in the pictures which were refreshed during the time of the exposure. (Note that other parts of the picture may also be visible, but not bright, due the phosphor persistence.) In Figure 10, only a small slice of the full picture is visible. From the size of the visible part, the exposure time can be calculated.

A simple approach to estimate the exposure time is the following: let us calculate the number N of rows on the taken picture; for this, the display should contain a carefully designed pattern containing horizontal lines, e.g., in every tenth row. The time $T_{line}$ necessary to draw one line can be calculated from the refresh rate and the number of lines of the monitor. A simple estimate of the exposure time can be the following:

(8) $$T_{exp}=N\cdot T_{line}.$$

**Figure 11:**
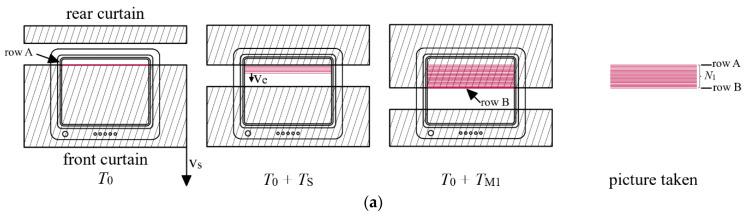

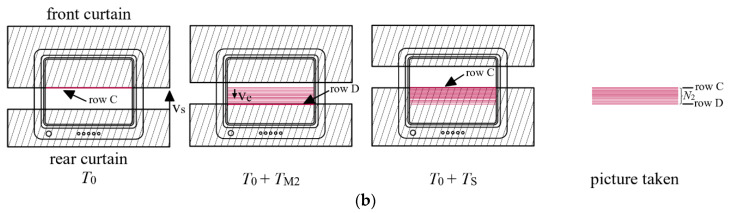
Exposure of a monitor screen. The refreshing of the monitor is done from top to bottom. (**a**) Inverted camera (in upside down position), curtain falling from top to bottom on the image. (**b**) Camera in normal position, curtains moving from bottom to up on the image.

This method, however, has bias, and may be significantly improved by investigating the process of exposure, as shown in Figure 11a. Let us suppose that the monitor is refreshed from top to bottom, i.e., first the uppermost row is drawn, then the next, until the last row at the bottom of the screen. Also let us suppose that the camera is placed so that the curtains fall in the same direction as the rows follow each other on the image (since cameras create inverted images, this happens if the camera is oriented upside down).

As shown in Figure 11a, the image is refreshed from top to bottom with speed $v_{e}$ rows per second, where $v_{e}=1\;\mathrm{row}/T_{line}$. For sake of convenience, let us define the speed of the curtains as $v_{c}$ rows per second (i.e., in one second the curtain would cover/uncover $v_{c}$ rows of the monitor in the taken picture), the speed vector $v_{c}$ pointing from top to bottom. Notice that in practice $v_{c}\gg v_{e}$.

At time instant $T_{0}$, the monitor draws row *A* and the camera’s front curtain just opens before row *A*: it will be the first row shown in the picture. Since the curtain is faster than the beam, the front curtain will uncover the area below row *A*, followed by the slower beam. At time instant $T_{0}+T_{exp}$, the rear curtain reaches row *A* and covers it. At time instant $T_{0}+T_{M1}$, the rear curtain reaches the actually refreshed row *B* and covers it. It is the last row shown in the picture. The picture contains rows between *A* and *B*, their number is denoted by $N_{1}$.

Notice that the number of lines refreshed during $T_{exp}$ is always smaller than $N_{1}$: the fact that the speed of the shutter is finite causes a bias in the measurement; the measured time according to (8) with $N=N_{1}$ is always longer than the real length of the exposure.

Notice that between time instants $T_{0}+T_{exp}$ and $T_{0}+T_{M1}$ the rear curtain covered the distance between rows *A* and *B*, thus $N_{1}$ rows. The beam covered the same distance between time instants $T_{0}$ and $T_{0}+T_{M1}$ Thus the following equation holds:

(9) $$N_{1}=v_{e}T_{M1}=v_{s}\left(T_{M1}-T_{exp}\right).$$

Let us consider the case when the camera is rolled 180 degrees (it is now in normal position), as shown in Figure 11b. Now, the vertical speed of the beam points downwards, while the speed of the curtains points upwards. The process of exposure is the following: At time instant $T_{0}$, the front curtain uncovers row *C*, which will be the first row shown in the picture. At time instant $T_{0}+T_{M2}$ the rear curtain reaches and covers the currently refreshed row *D*. This will be the last row shown in the picture. Some time later, at $T_{0}+T_{exp}$, the rear curtain reaches the position of row *C*. In the taken picture rows between *C* and *D* are shown, their numbers being $N_{2}$.

Now let us notice that the rear curtain between time instants $T_{0}+T_{M2}$ and $T_{0}+T_{exp}$ covered the distance between rows *C* and *D*, altogether $N_{2}$ rows. The same distance was covered by the beam between time instants $T_{0}$ and $T_{0}+T_{M2}$; thus, the following equation can be constructed:

(10) $$N_{2}=v_{e}T_{M2}=v_{s}\left(T_{exp}-T_{M2}\right).$$

Note that in this case there is a bias, too, if the naïve approach of (8) is used, but now the measured time is always smaller than $T_{exp}$.

From (9) and (10) the unbiased estimate of $T_{exp}$ can be expressed, as follows:

(11) $$T_{exp}=\frac{2T_{M1}T_{M2}}{T_{M1}+T_{M2}}=\frac{2}{v_{e}}\frac{N_{1}N_{2}}{N_{1}+N_{2}}.$$

The uncertainty of the estimated exposure time can be calculated as follows. Using the partial derivatives $\frac{\delta T_{exp}}{\delta T_{M1}}=\frac{2T_{M2}^{2}}{{\left(T_{M1}+T_{M2}\right)}^{2}}$ and $\frac{\delta T_{exp}}{\delta T_{M2}}=\frac{2T_{M1}^{2}}{{\left(T_{M1}+T_{M2}\right)}^{2}}$ of (11), the variation of $T_{exp}$, in the presence of measurement uncertainties $\Delta T_{M1}$ and $\Delta T_{M2}$, can be approximated as follows:

(12) $$\Delta T_{exp}≅\frac{\delta T_{exp}}{\delta T_{M1}}\Delta T_{M1}+\frac{\delta T_{exp}}{\delta T_{M2}}\Delta T_{M2}=\frac{2T_{M2}^{2}}{{\left(T_{M1}+T_{M2}\right)}^{2}}\Delta T_{M1}+\frac{2T_{M1}^{2}}{{\left(T_{M1}+T_{M2}\right)}^{2}}\Delta T_{M2}.$$

If the reading uncertainties $\Delta T_{M1}$ and $\Delta T_{M2}$ are maximized by ΔT, i.e.,

(13) $$\Delta T≅\max\left(\Delta T_{M1}\right)≅\max\left(\Delta T_{M2}\right),$$

then the maximum estimation uncertainty $\Delta T_{exp,\;max}$ is the following:

(14) $$\Delta T_{exp,\;max}=2\frac{T_{M1}^{2}+T_{M2}^{2}}{{\left(T_{M1}+T_{M2}\right)}^{2}}\Delta T,$$

and the maximum relative uncertainty is

(15) $$\frac{\Delta T_{exp,\;max}}{\Delta T_{exp}}=\frac{T_{M1}^{2}+T_{M2}^{2}}{T_{M1}T_{M2}\left(T_{M1}+T_{M2}\right)}\Delta T.$$

Using approximate value $T_{M}≅T_{M1}≅T_{M2}$, the maximum relative uncertainty is estimated as follows:

(16) $$\frac{\Delta T_{exp,\;max}}{\Delta T_{exp}}≅\frac{\Delta T}{T_{M}}=\frac{\Delta N}{T_{exp}v_{e}}.$$

**Figure 12:**
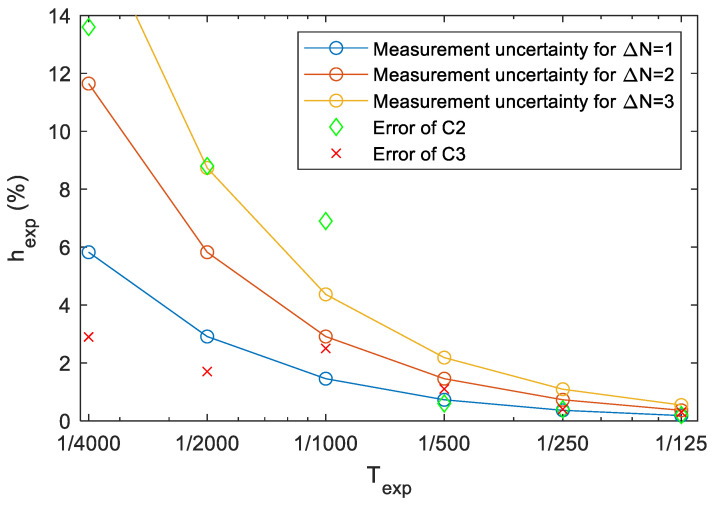
Maximum theoretical relative uncertainty of the exposure time estimates of the monitor measurement. Measurement errors of C2 and C3, using the unbiased estimate of (11), are also shown.

Since $v_{e}$ is constant, and the reading uncertainty ΔN is also approximately constant (1–3 lines of uncertainty was experimented during the measurements), the relative estimation uncertainty (16) is inversely proportional with the exposure time. Figure 12 shows the theoretical relative uncertainty, for ΔN=1,2,3. Thus, reasonable measurements are possible between 1/125 and 1/4000; the uncertainty of the estimation may be below 1% above 1/500, but in the short exposure time region it can be as high as 10%.

**Figure 13:**
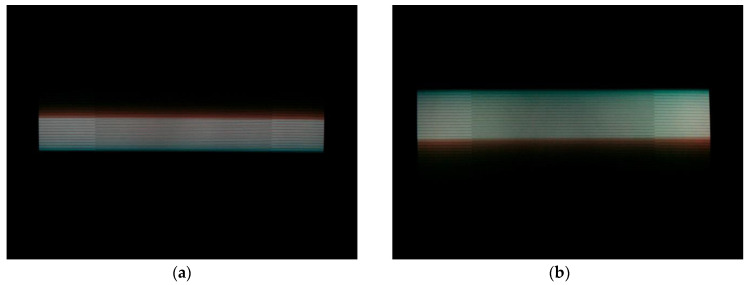
Example measurements of C2 with a monitor screen with 1/500 s. (**a**) camera in normal position, showing 120 lines. (**b**) camera upside-down, showing 163 lines.

An example measurement of C2 is shown in Figure 13, with exposure time of 1/500 sec. The results were $N_{1}=120$ and $N_{2}=163$, in normal and upside-down camera positions, respectively. The monitor draws 1 line in 14.56 μs ($v_{e}=1/14.56\;\mathrm{lines}/\mu s$), thus the naïve measurement results, according to (8) are 1.75 ms and 2.37 ms. The unbiased estimate of (11) is 2.01 ms, which is a good estimate of the nominal 1/500 s = 2 ms value.

More measurement results are shown in Table 4, the errors are also plotted on Figure 12, for cameras C2 and C3. Since C3 has global shutter, for this camera $N_{1}=N_{2}$, so either approach gives the same unbiased estimate. The measurement results are quite close to the nominal values, for longer exposure time with error below 1%, while for shorter exposure times the error increased to 3%, possibly due to measurement inaccuracies, as was expected according to (16). C2 has rolling shutter, thus the naïve approach resulted in high errors. Notice that the error is always negative in normal, while positive in upside down position. The unbiased estimator shows good agreement between 1/125 s and 1/500 s, but there are significant differences for shorter shutter times (higher than the expected measurement uncertainties), thus the timing of the camera is probably not accurate in this range.

### 4.3. Running LED Array

**Figure 14:**
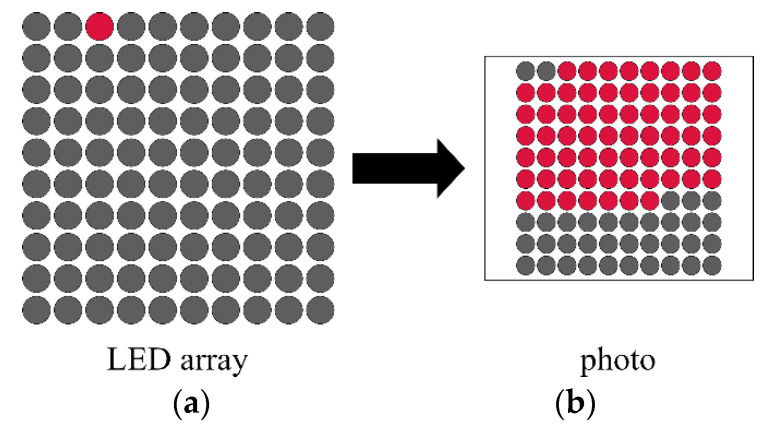
Measuring exposure time with a running *LED* on an *LED* array. (**a**) One *LED* is on at a time (**b**) On the exposed image multiple *LED*s are bright.

The moving object can be replaced by an *LED* array: in this setup, as shown in Figure 14, one *LED* is switched on at a time for time $T_{ON}$. The *LED*s light up one after another, creating an effect as if one *LED* was running circularly along the array. Such devices may use *LED* stripes, as in [38], where stripes of 100 *LED*s were proposed, while the commercial product [39] utilizes a 10 × 10 array of *LED*s.

Trivially, if a picture contains $N_{ON}$ bright *LED*s, then the exposure time $T_{exp}$ can be calculated as follows:

(17) $$T_{exp}=N_{ON}\cdot T_{ON}.$$

Notice that the array setup, shown in Figure 14, results in the same problem that was discussed in the CRT case: the final speed of the rolling shutter will cause a bias. When the *LED*s are arranged in a single row, this effect is not present.

In practice the first and last *LED*s in the bright series may not be as bright as the other ones. Although the observed light intensity could be used to refine the estimate, it is safer to state that the reading uncertainty is not more than ±2 in the count of $N_{LED}$. The timing inaccuracy of the *LED*s can be neglected, thus the relative uncertainty of the measurement can be estimated as

(18) $$h_{exp}=\frac{2T_{ON}}{T_{exp}}=\frac{2}{N_{ON}}.$$

**Figure 15:**
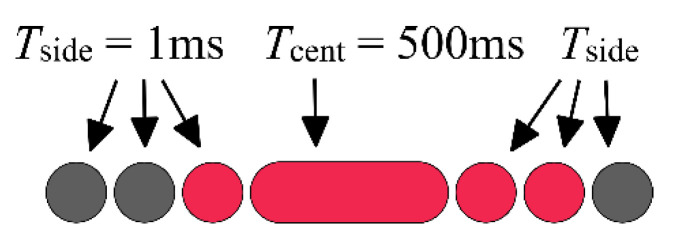
LED array using multiple timers.

Due to the limited resolution, the best result that can be obtained, according to (18), is 2/$N_{TOTAL}$, where $N_{TOTAL}$ is the total number of *LED*s in the device. In a device containing 100 *LED*s the relative uncertainty would be around 2%. The resolution, thus the accuracy, can be improved using multiple *LED* timers, as shown in Figure 15.

In the multi-timer device, the central *LED*, illustrated as a wider *LED*, has on-time $T_{CENT}$, while the on-time of the side *LED*s is $T_{SIDE}$. Notice that $T_{CENT}$ may be significantly higher than $T_{SIDE}$. In the illustration $T_{SIDE}=1\;\mathrm{ms}$ and $T_{CENT}=500\;\mathrm{ms}$. If the central *LED* is bright, on the left side there is $N_{LEFT}=1$ bright *LED*, and on the right side there are $N_{RIGHT}=2$ bright *LED*S, as shown in Figure 15, then the exposure time is

(19) $$T_{exp}=T_{CENT}+\left(N_{LEFT}+N_{RIGHT}\right)T_{SIDE}{,}_{}$$

resulting in $T_{exp}=503\;\mathrm{ms}$. Notice that the resolution is now determined by $T_{SIDE}$, which may be a very small value, providing high resolution and high accuracy with a small number of *LED*s. The uncertainty is now estimated as

(20) $$h_{exp}=\frac{2T_{SIDE}}{T_{exp}}.$$

which in the example of Figure 15 results in an error of 0.4%.

When the multi-timer device is used, for an unknown exposure time usually an iterative approach is necessary: first, the approximate exposure time $T_{exp}$ is determined with $T_{CENT}=T_{SIDE}$, then $T_{SIDE}$ is reduced and $T_{CENT}$ is set so that $T_{CENT}+4T_{SIDE}\approx T_{exp}$. Using the more and more accurate estimate $T_{exp}$, the values of $T_{CENT}$ and $T_{SIDE}$ are updated using smaller and smaller $T_{SIDE}$ values, until the required resolution is reached.

The utilization of a device, similar to Figure 15, has disadvantages, too. Notice that the *LED* pattern must fulfill the following requirements, R1 and R2, in order to contain meaningful measurement:

R1: The leftmost and rightmost *LED*s must be dark (otherwise the numbers $N_{LEFT}$ or $N_{RIGHT}$ would not be meaningful)
R2: The two side *LED*s, next to the central *LED* must be bright (otherwise it would not be sure that the central *LED* was on for the full time of $T_{CENT}$)

To capture such pattern, either the camera must be synchronized to the measurement device, or the user must be really lucky: the higher the ratio of $T_{CENT}/T_{SIDE}$ the less probable that the image satisfies the requirements. If camera synchronization is not possible but the camera is able to record video, an alternative ‘quasi-synch’ method can be used, as follows:

The running *LED* is not cycling continuously: the *LED* runs along the line once and stops at the last *LED*. The cycle will start again so that the repeat time of the cycle is $T_{rep}$. If the camera’s framerate is $f_{frame}$ then $T_{rep}$ is tuned around $1/f_{frame}$:

(21) $$T_{rep}=\frac{1}{f_{rame}}-\Delta T_{rep},$$

For $\Delta T_{rep}=0$, the camera and the running *LED*s would be perfectly synchronized, thus pictures of the running *LED* would be taken at exactly the same phase and all pictures of the video would contain the same image. Instead, $\Delta T_{rep}≅T_{SIDE}$ is used during the measurement, when each picture of the video is taken at a phase $\Delta T_{rep}$ time later than the previous one. Thus, the captured video stream scans the running *LED* sequence, with offset changing by steps of $\Delta T_{rep}$ in each frame, eventually catching a desired time instant, similar to Figure 15. After the video recording, a suitable frame, satisfying requirements R1 and R2, is selected and $N_{LEFT}$ and $N_{RIGHT}$ are measured on the frame. Finally, (19) is used to calculate the exposure time estimate. Notice that the “quasi-synch” method in fact uses equivalent sampling [40,41], which will be discussed in Section 5.

**Figure 16:**
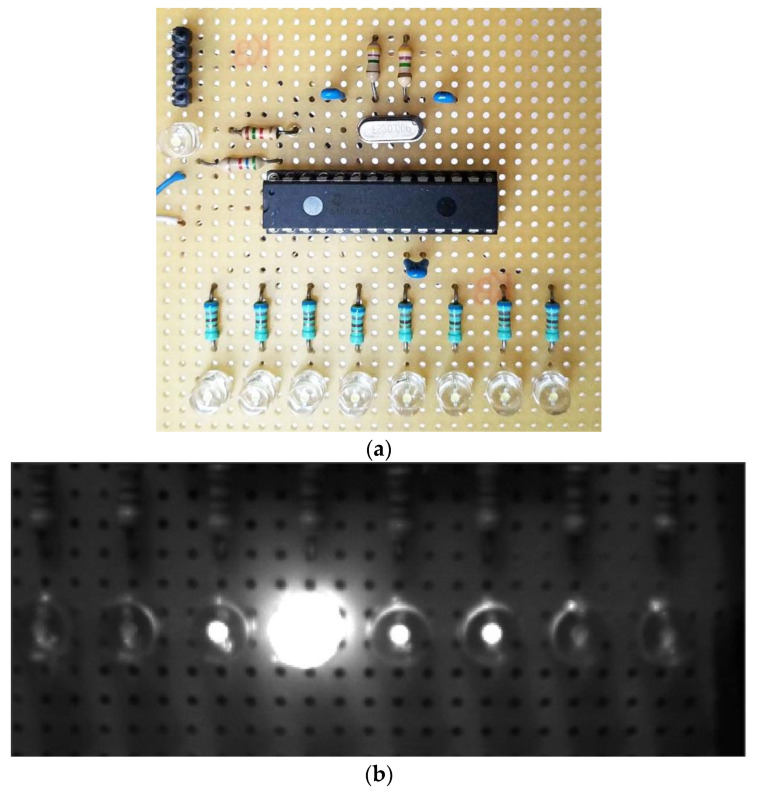
(**a**) *LED* array equipment. (**b**) Measurement of camera C3, with nominal $T_{exp}=1/1000$ and settings $T_{CENT}=1005\;\mu s$, $T_{SIDE}=1\;\mu s$.

Figure 16 shows the multi-timer measurement equipment and a photo taken by C3 with settings $T_{CENT}=1005\;\mu s$, $T_{SIDE}=1\;\mu s$. From Figure 13b, the values $N_{LEFT}=1$ and $N_{RIGHT}=2$ can be read, thus, according to (19), the measured exposure time is $T_{exp}=1008\;\mu s$.

Measurement results of C3 can be seen in Table 5. The nominal value, set by the user, is internally rounded and slightly modified by the camera, and the exact value can be queried. Thus, the column *Reported exposure time* shows the exact timing reported by the camera. Column $T_{exp}$ shows the estimated exposure times, along with the maximum uncertainty. The uncertainty values were calculated as $2T_{SIDE}$; e.g., 1004±2 μs means that the side *LED*s on-time was 1 μs, causing maximum 2 μs reading uncertainty. As the results show, there is a systematic error of approximately 6 μs, which is especially visible at the lower time region: the camera has higher exposure time than it is actually reported by the camera’s software. Similar effects were observed concerning other camera types of the same manufacturer [42]. The last column *Relative error^(br)^* shows the relative measurement error, after the 6 μs bias was subtracted from the reported values. The accuracy of the measurement is really good: at lower speed the relative error is way below 1%, while around the few microseconds range the error increased to 7%. This measurement method allows exposure time measurement even with 1 μs accuracy.

## 5. Measurement Using Equivalent Sampling

**Figure 17:**
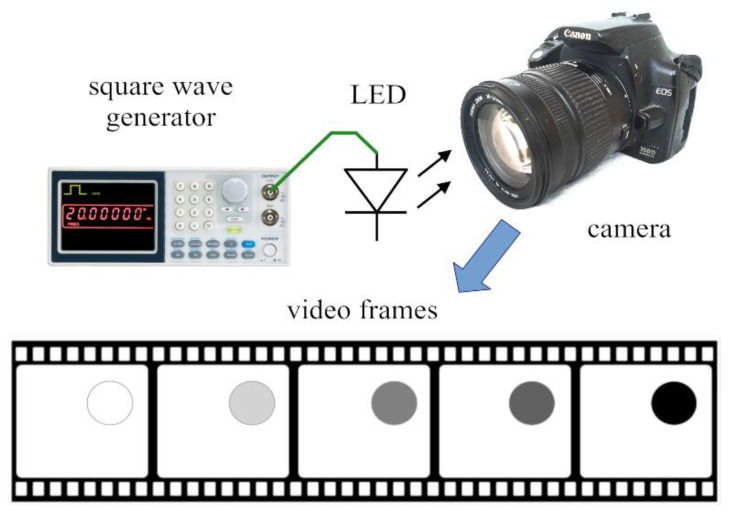
The equivalent sampling-based measurement method.

A completely different approach was proposed in [42] for digital cameras having video mode. The method is illustrated in Figure 17. The camera is used in video mode, i.e., it captures frames with period $T_{CAM}$, where $T_{CAM}=1/f_{frame}$ and $f_{rame}$ is the frame rate, e.g., 30 FPS (frames per second). The input is a blinking light, produced by an *LED*, driven by a symmetrical square wave, with period $T_{LED}$. With properly chosen blinking frequency, the camera will record a slowly blinking *LED*. The intensity function of the recorded *LED* is utilized to compute the exposure time.

**Figure 18:**
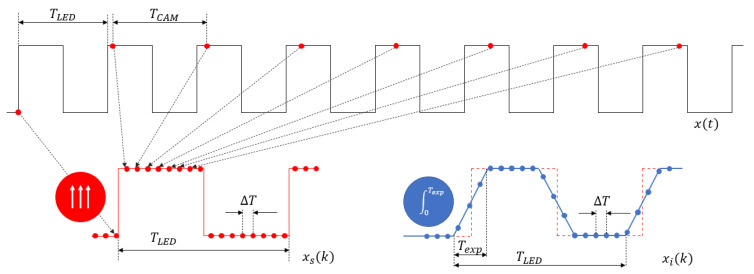
Operation of the equivalent sampling-based measurement method. Black rectangle signal: intensity of the blinking *LED*. Red dots: samples using ideal (impulse) sampling. Blue dots: samples using integral sampling with integral time of $T_{exp}$.

The operation is illustrated in Figure 18. The blinking frequency is selected so that $T_{CAM}$ is approximately, but not exactly the multiple of $T_{LED}$:

(22) $$T_{CAM}=nT_{LED}+\Delta T,$$

where $\Delta T\ll T_{LED}$ and n≥1 integer. For a moment, let us suppose that we sample the *LED* signal with ideal (impulse) sampling, as shown in Figure 18 in red dots. Let us denote the original signal by x(t), and the sampled signal by $x_{s}\left(k\right)=x\left(kT_{CAM}\right)$. Notice that due to (22), consecutive samples will have the following properties:

(23) $$x_{s}\left(k\right)=x\left(kT_{CAM}\right)\;x_{s}\left(k+1\right)=x\left(\left(k+1)T_{CAM}\right)\right)=x\left(kT_{CAM}+nT_{LED}+\Delta T\right)=x\left(kT_{CAM}+nT_{LED}+\Delta T\right).$$

Because of the periodicity of x(t), $x\left(t+nT_{LED}\right)=x\left(t\right),$ for any integer n, thus

(24) $$x_{s}\left(k+1\right)=x\left(kT_{CAM}+\Delta T\right).$$

According to (24), it seems that sample *k* + 1 is taken ΔT time after sample *k*. It is exactly the principle of equivalent sampling: a periodic signal x(t) is sampled with low sampling frequency $1/T_{CAM}$ but the sampled signal is the same as if x(t) was sampled with high frequency 1/ΔT, as shown in Figure 18 [40].

Cameras, however, do not use ideal sampling, rather the operation of light sensors (both films and electronic sensors) can be modelled as integrators: the camera integrates the incoming light for the length of exposure. Thus, the real sample $x_{i}\left(k\right)$, taken by the camera and shown with blue dots in Figure 18, is computed by the integral of x(t), between time instants $kT_{CAM}$ and $kT_{CAM}+T_{exp}$, as follows:

(25) $$x_{i}\left(k\right)=\overset{kT_{CAM}+T_{exp}}{\underset{kT_{CAM}}{\int }}x\left(t\right)dt.$$

The integral of a symmetrical square wave is a symmetrical trapezoid, where the lengths of the rising and falling edges are $T_{exp}$, as shown in Figure 18.

Let us denote the number of samples on the rising (or falling) edge by $N_{exp}$, and the number of samples in the full period by $N_{LED}$. Thus, the following approximate equations hold:

(26) $$T_{exp}≅N_{exp}\Delta T,\;T_{LED}≅N_{LED}\Delta T.$$

From (26) the exposure time estimate can be calculated as follows:

(27) $$T_{exp}=T_{LED}\frac{N_{exp}}{N_{LED}}.$$

The exposure time measurement is performed as follows:

Step 1. The generator’s period length $T_{LED}$ is set according to (22), using any integer number n.
Step 2. The output of the camera is observed and $T_{LED}$ is fine-tuned so that the video stream shows a slowly blinking *LED*. The period length may be several seconds or even minutes. After the tuning the value of $T_{LED}$ is read.
Step 3. A sufficiently long record is gathered (at least one full period)
Step 4. One pixel of the *LED* (preferably at the center of the screen) is selected and the intensity function of this pixel as a function of time is used.
Step 5. The number of samples $N_{exp}$ on the rising (or falling) edge is counted.
Step 6. The number of samples $N_{LED}$ in the full period is counted.
Step 7. The exposure time is estimated using (27).

The uncertainty of $T_{LED}$ can be neglected, when a good quality oscillator is used, thus the uncertainty of the measurement can be estimated from (27), using the partial derivatives $\frac{\delta T_{exp}}{\delta N_{exp}}$ and $\frac{\delta T_{exp}}{\delta N_{LED}}$, as follows:

(28) $$\Delta T_{exp}=\frac{\delta T_{exp}}{\delta N_{exp}}\Delta N_{exp}+\frac{\delta T_{exp}}{\delta N_{LED}}\Delta N_{LED}=\frac{T_{exp}}{N_{exp}}\Delta N_{exp}-\frac{T_{exp}}{N_{LED}}\Delta N_{LED}=T_{exp}h_{Nexp}-T_{exp}h_{NLED},$$

thus, the relative uncertainty of $T_{exp}$ is the following:

(29) $$h_{exp}=\frac{\Delta T_{exp}}{T_{exp}}=h_{Nexp}-h_{NLED}.$$

The reading uncertainty depends on the measurement noise, and can be enhanced e.g., using linear regression [42]. In our test environment, the reading uncertainty was 2–5 samples. According to (28) and (29), the higher the number of measurements ($N_{1}$ and $N_{2}$) the better the accuracy, thus for high quality measurements the $T_{LED}$ blinking period must be tuned so that the blinking period on the image is long enough. An example is provided to illustrate the determination of measurement parameters, given the accuracy needs.

Let us suppose that the exposure time to be measured is approximately $T_{exp}≅1/1000$ s, and we want to determine its exact value with 1% accuracy. The camera’s sampling interval is $T_{CAM}=1/30$ s, parameter n=5, thus, according to (22), $T_{LED}≅1/150$ sec. If the reading accuracy $\Delta N_{exp}≅\Delta N_{NLED}≅2=\Delta N$ samples then, according to (29), the accuracy requirement can be written as $h_{exp}≅\Delta N\left(\frac{1}{N_{exp}}+\frac{1}{N_{LED}}\right)<1\%.$ From (27), $\Delta N_{exp}=\Delta N_{LED}\frac{T_{exp}}{T_{LED}}=\frac{\Delta N_{LED}}{6.7}$, thus the accuracy requirement becomes $2\left(\frac{6.7}{N_{LED}}+\frac{1}{N_{LED}}\right)<1\%$, from which $N_{LED}>$ 1533. Thus, one blinking period must contain at least 1533 samples, which means that $T_{LED}$ time in Step 2 must be tuned until the observed blinking period is longer than $1533\cdot \frac{1}{30}s=51\;s$.

**Figure 19:**
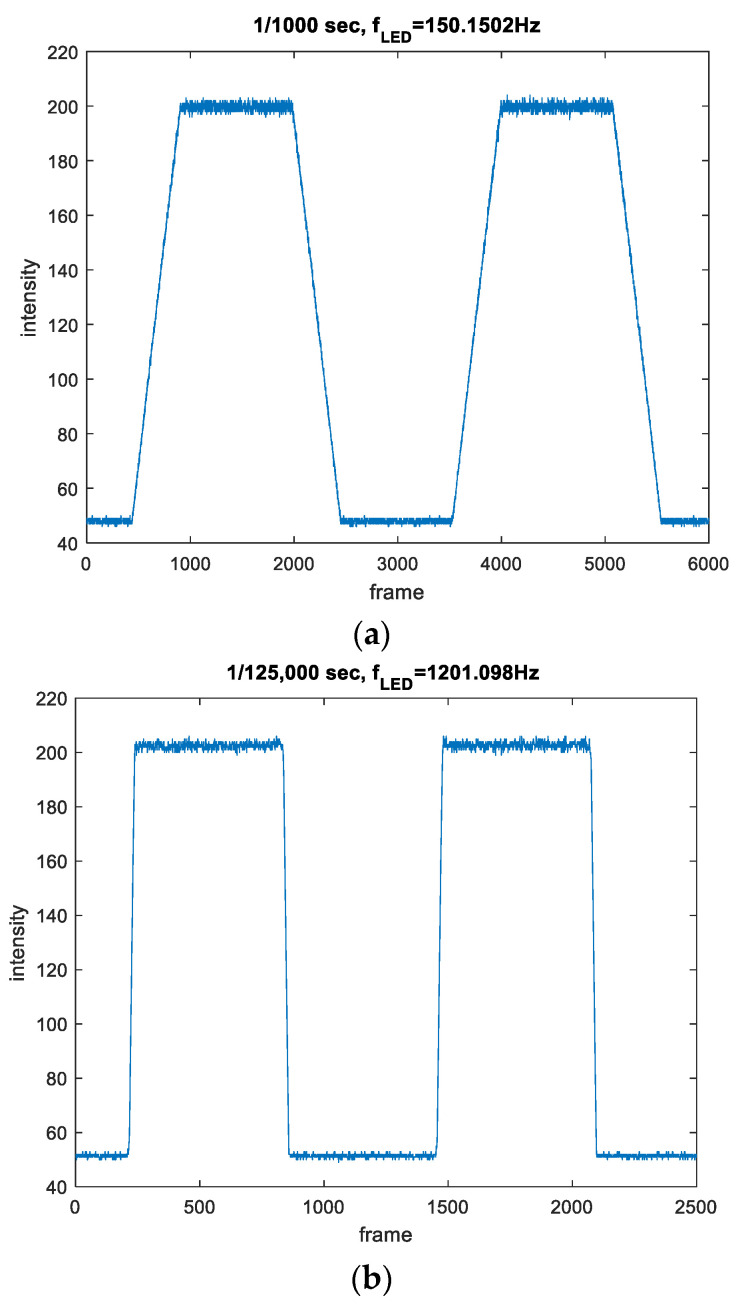
Measurements of camera C3 with equivalent sampling. (**a**) 1/1000 s, (**b**) 1/125,000 s.

Two example measurements, with nominal exposure times of 1/1000 and 1/125,000, are detailed in Table 6, using camera C3. The table contains the exposure times reported by the camera, the counted values $N_{exp}$ and $N_{LED}$, the frequency $f_{LED}$ of the *LED*, the estimated exposure time according to (27), and the estimated maximum relative uncertainty $h_{exp}$, according to (29). Figure 19 shows the plots of the corresponding measurements.

The maximum estimation uncertainty $h_{exp}$ was calculated using counting uncertainty $\Delta N_{exp}≅\Delta N_{NLED}≅2$. For the first case, using (29), the maximum relative measurement uncertainty is $h_{exp}≅\frac{2}{233}+\frac{2}{1540}≅1\%$. In the second case the maximum relative uncertainty is $h_{exp}≅\frac{2}{23}+\frac{2}{1239}≅9\%$.

The measurement results of C3 are summarized in Table 7. In addition to the reported exposure time, the table also contains the corrected exposure times, due to the systematic bias discussed in Section 4.3. Values in column *Relative error* are calculated with respect to the reported nominal values, while column *Relative error^(br)^* shows the error with respect to the corrected (unbiased) values. The measured exposure times correspond very well with the corrected values, the relative error growing above 1% only in case of the very short exposure times.

## 6. Comparison and Evaluation

In this section the reviewed measurement methods are summarized and compared. Table 8 summarizes the main features of the discussed methods.

The direct method is applicable only for cameras where the focal plane is accessible, i.e., film cameras, or cameras where the camera frame can be opened. The method is also suitable for testing during manufacturing or servicing. The method can be applied for exposure times longer than 1/10,000. The minimum exposure time that can be measured is limited by the measurement noise. The measurement uncertainty at the shorter times may be as high as 10%, but as the exposure time increases the measurement uncertainty decreases to approx. 0.5%. The measurement process is simple: only one exposure is required, followed by a simple time measurement on the oscilloscope. No special equipment is required: a light source, a photosensor and an oscilloscope is necessary. The direct method can be used to measure the exposure time according to the standard at the center of the picture frame, or alternatively the measurement can be made at any point of the picture frame.

The turntable method (or any alternative blur-based method using a moving physical object) is a simple method requiring only a single shot with any type of camera. The measurement range is quite narrow, from 1/125 sec to 2 s: at short exposure times the angle to be measured is very small, resulting in poor accuracy, while at long exposure times the angle would be higher than 360 degrees, which cannot be detected. Thus, at shorter times the uncertainty is high (even 10%), but at longer times uncertainty around 1% can be reached.

The method using a monitor has a somewhat wider measurement range, from 1/10,000 to 1/125. The measurement process is simple, in general requiring two exposures (one in case of global shutters). Here the measurement range is limited by the fact that at short exposure times the number of rows is small (possibly fractional), causing large detection uncertainties, while at long exposure times the exposed rows fill the full monitor, prohibiting the measurement. The accuracy is modest at short exposure times but can be better than 1% at longer times. The application of the method is more and more difficult since CRT monitors in good operating condition are hard to find.

The running *LED* method with single timer requires an *LED* array with large number of *LED*s. The commercial equipment is quite expensive. The accuracy in the full operation range is good, around a few percent. Here the accuracy is limited by the detection error, which is uniformly around 1–2 *LED*s, independently of the measurement range. The measurement process is simple, only one exposure is required, followed by the counting of the bright *LED*s, which can be automatized. This is a general and comfortable method, suitable for most requirements.

The multiple timer version of the running *LED* method offers a much simpler measurement device and potentially much higher accuracy, at a price of more complicated measurement process. The measurement may require multiple iterations, until the required precision is reached. Moreover, either the camera needs to be synchronized to the measurement device, or a video-based sampling is required, in order to provide a picture containing the necessary information to calculate the shutter time. This method is suitable for very high accuracy measurements. The measurement range at very small exposure times is practically limited by the minimal timing of the side *LED*s.

The accuracy of the equivalent sampling-based method is also excellent, similarly to the multi-timer *LED*. The measurement equipment is very simple, containing only a signal generator and an *LED*. The measurement process requires the tuning of the generator frequency, by observing the under-sampled camera output. The measurement process may require several minutes in order to gather the necessary amount of data. This method is applicable only for cameras with video mode. The range of measurable exposure times is limited by the detection error of the equivalent period length, allowing measurements in the microsecond range with modest accuracy, but for longer exposure times very high precision can be achieved.

**Figure 20:**
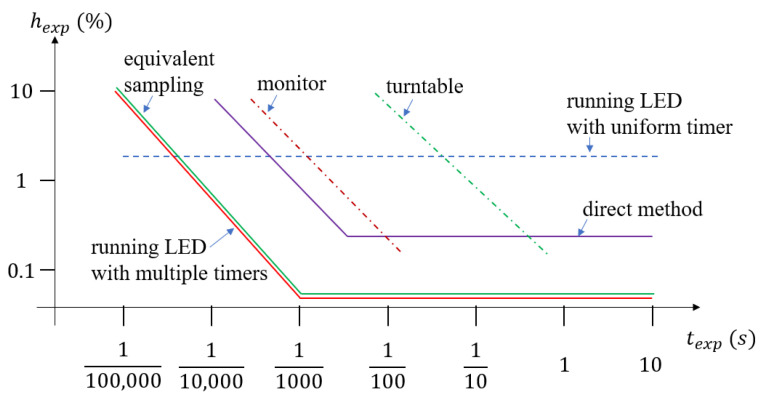
Accuracy and regions of applicability of the discussed methods.

The applicable measurement regions, along with the achievable accuracy, for all methods are summarized in Figure 20.

## 7. Conclusions

This paper reviewed several methodologies and measurement devices to measure the exposure time of cameras. The direct method, several motion blur methods, and the equivalent sampling method were discussed, along with the investigation of their performance properties. All methods were illustrated by real measurement examples.

The direct method is applicable for cameras, where the focal plane is accessible. Its accuracy may be better than 1%, for exposure time higher than 1/100 s. The turntable and monitor based methods have modest accuracy and much narrower range, from 1/10,000 s to 1/100 s and 1/100 s to 1 s, respectively. The running *LED* with uniform timer method has a uniformly excellent performance, with a few percent uncertainty, starting from exposure times even as low as 1/100,000 s. The running *LED* with multiple timers and the equivalent sampling methods provide wide measurement ranges from 1/100,000 s and can provide excellent precision, with estimation uncertainties well below 1%.

## Figures and Tables

**Table 1:** Shutter properties of the cameras used for testing in the paper.

	C1	C2	C3
Shutter type	mechanical	electromechanical	electronic
Rolling/global	rolling	rolling	global
Shutter time range	1/500–1/30 s	1/4000–30 s	1/125,000–31.9 s
Focal plane available	yes	no	no
Video mode	no	no	yes

**Table 2:** Measurement results of C1, with the direct measurement.

Nominal Exposure Time (s)	Measured Exposure Time (ms)	Relative Error (%)
1/30	23.4	−29.8
1/60	14.34	−14.0
1/125	7.37	−7.9
1/250	4.15	3.75
1/500	1.94	−3.0

**Table 3:** Turntable measurement results for C2 and C3.

Nominal Exposure Time	C2		C3	
	$T_{exp}$ (μs)	Relative Error (%)	$T_{exp}$ (μs)	Relative Error (%)
1/1	978,492	−2.1	1,001,986	0.5
1/2	494,514	−1.1	495,435	−0.9
1/4	242,970	−2.8	250,896	0.4
1/8	127,179	1.7	123,449	−1.2
1/15	65,129	−2.3	63,894	−4.2
1/30	33,037	−0.9	32,221	−3.4
1/60	13,679	−17.9	15,789	−5.2
1/125	6772	−15.4	7072	−11.6

**Table 4:** Measurement results of the CRT method.

Nominal Exposure Time	C3		C2					
	Using either (7) or (10)		Using (10)		Using (7), Normal Camera Position		Using (7), Camera Upside Down	
	$T_{exp}$ (μs)	Rel. Error (%)	$T_{exp}$ (μs)	Rel. Error (%)	$T_{exp}$ (μs)	Rel. Error (%)	$T_{exp}$ (μs)	Rel. Error (%)
1/125	7979	−0.3	7983	−0.2	6873	−14	9523	19
1/250	3909	−0.4	3982	−0.4	3407	−15	4791	20
1/500	1980	−1.1	2013	0.6	1747	−13	2373	19
1/1000	976	−2.5	1069	6.9	917	−8	1281	28
1/2000	495	−1.7	544	8.8	451	−10	684	37
1/4000	248	2.9	284	13.6	233	−7	364	46

**Table 5:** Measurement results of C3, using the multi-timer running *LED* method.

Nominal Exposure Time (s)	Reported Exposure Time (μs)	$T_{exp}$ $\;(\mu s)\;\pm \;2T_{SIDE}$	Relative Error (%)	Relative Error^(br)^ (%) (Bias Removed)
1/60	16,667	16,665 ± 10	−0.01	−0.05
1/125	8000	8004 ± 4	0.05	−0.03
1/250	4004	4008 ± 4	0.1	−0.05
1/500	2002	2006 ± 4	0.2	−0.1
1/1000	1001	1008 ± 2	0.6	0.1
1/2000	496	503 ± 2	1.4	0.2
1/4000	252	259 ± 2	2.8	0.4
1/10,000	98	104 ± 2	6.1	0
1/20,000	49	56 ± 2	14.3	1.8
1/125,000	8	15 ± 2	87.5	7.1

**Table 6:** Parameters of two example measurements of C3, using the equivalent sampling method.

Reported Exposure Time	$N_{exp}$	$N_{LED}$	$f_{LED}$	Estimated Exposure Time (27)	$h_{exp}\;\;(29)$
1001 μs	233	1540	150.15 Hz	1007.6 μs	1%
8.1 μs	23	1239	1201.098 Hz	15.5 μs	9%

**Table 7:** Measurement results of C3, using the equivalent sampling method.

Nominal Exposure Time	Reported Exposure Time	Exposure Time with Bias Removed	$T_{exp}$ (μs)	Relative Error (%)	Relative Error^(br)^ (%)
1/60	16,667	16,673	16,650	−0.1	−0.1
1/125	8000	8006	8002	0.03	−0.1
1/250	4004	4010	4005	0.03	−0.1
1/500	2002	2008	2008	0.3	0
1/1000	1001	1007	1005	0.4	−0.2
1/2000	496	502	502	1.2	0
1/4000	252	258	259	2.8	0.4
1/10,000	98	104	104	6.1	0
1/20,000	49	55	56	12.2	1.8
1/125,000	8	14	15	87.5	7.1

**Table 8:** Comparison of the discussed methods.

	Direct Method	Turntable	Monitor	Running *LED* Uniform Timer	Running *LED* Multi Timer	Equivalent Sampling
Applicability	film cameras, manufacturing	any camera	any camera	any camera	cameras with synchronization or video	video
Meas. range (s)	1/10,000 <	1/125–2	1/10,000–1/125	1/100,000<	1/100,000<	1/100,000<
$\mathrm{uncertainty}\;\mathrm{for}\;\mathrm{short}\;T_{exp}$	≅10%	≅10%<	≅10%	1–3%	1–10%	1–10%
$\mathrm{uncertainty}\;\mathrm{for}\;\mathrm{long}\;T_{exp}$	≅1%	≅1%	≅1%	1–3%	<<1%	<<1%
Meas. time	1 exposure	1 exposure	2 exposures	1 exposure	minutes (iterative, video)	minutes (freq. tuning, video)
Equipment cost	high	low	low	high	medium	medium
Measurement complexity	low	low	medium	low	high	medium
Pros	fast, simple	simple	simple, moderate range	fast, accurate, simple, wide range	inexpensive, very accurate, wide range	inexpensive, very accurate, wide range
Cons	opening of camera frame is necessary	narrow range, modest accuracy	obsolete technology (CRT)	expensive equipment	long and cumbersome measurement	long measurement, video only

## Data Availability

Not applicable.

## References

[1] Kelby S. (2020). The Digital Photography Book.

[2] Tanizaki K., Tokiichiro T. A real camera interface enabling to shoot objects in virtual space. Proceedings of the International Workshop on Advanced Imaging Technology (IWAIT).

[3] Banterle F., Artusi A., Debattista K., Chalmers A. (2017). Advanced High Dynamic Range Imaging.

[4] Várkonyi-Kóczy A.R., Rövid A., Hashimoto T. (2008). Gradient-Based Synthesized Multiple Exposure Time Color HDR Image. IEEE Trans. Instrum. Meas..

[5] McCann J.J., Rizzi A. (2012). The Art and Science of HDR Imaging.

[6] Gnanasambandam A., Chan S.H. (2020). HDR Imaging with Quanta Image Sensors: Theoretical Limits and Optimal Reconstruction. IEEE Trans. Comput. Imaging.

[7] Psota P., Çubreli G., Hála J., Šimurda D., Šidlof P., Kredba J., Stašík M., Lédl V., Jiránek M., Luxa M. (2021). Characterization of Supersonic Compressible Fluid Flow Using High-Speed Interferometry. Sensors.

[8] Wu T., Valera J.D., Moore A.J. (2011). High-speed, sub-Nyquist interferometry. Opt. Express.

[9] Beckwith S.V., Stiavelli M., Koekemoer A.M., Caldwell J.A., Ferguson H.C., Hook R., Lucas R.A., Bergeron L.E., Corbin M., Jogee S. (2006). The Hubble Ultra Deep Field. Astron. J..

[10] Feltre A., Bacon R., Tresse L., Finley H., Carton D., Blaizot J., Bouché N., Garel T., Inami H., Boogaard L.A. (2018). The MUSE Hubble Ultra Deep Field Survey-XII. Mg II emission and absorption in star-forming galaxies. Astron. Astrophys..

[11] Borlaff A., Trujillo I., Román J., Beckman J.E., Eliche-Moral M.C., Infante-Sáinz R., Lumbreras-Calle A., De Almagro R.T., Gómez-Guijarro C., Cebrián M. (2019). The missing light of the Hubble Ultra Deep Field. Astron. Astrophys..

[12] Wang S., Xu Y., Zheng Y., Zhu M., Yao H., Xiao Z. (2019). Tracking a Golf Ball with High-Speed Stereo Vision System. IEEE Trans. Instrum. Meas..

[13] Gyongy I., Dutton N.A.W., Henderson R.K. (2018). Single-Photon Tracking for High-Speed Vision. Sensors.

[14] Li J., Long X., Xu D., Gu Q., Ishii I. (2020). An Ultrahigh-Speed Object Detection Method with Projection-Based Position Compensation. IEEE Trans. Instrum. Meas..

[15] Cortés-Osorio J.A., Gómez-Mendoza J.B., Riaño-Rojas J.C. (2018). Velocity Estimation from a Single Linear Motion Blurred Image Using Discrete Cosine Transform. IEEE Trans. Instrum. Meas..

[16] Ma B., Huang L., Shen J., Shao L., Yang M., Porikli F. (2016). Visual Tracking Under Motion Blur. IEEE Trans. Image Processing.

[17] Zhang Y., Wang C., Maybank S.J., Tao D. (2021). Exposure Trajectory Recovery from Motion Blur. IEEE Trans. Pattern Anal. Mach. Intell..

[18] Saha N., Ifthekhar M.S., Le N.T., Jang Y.M. (2015). Survey on optical camera communications: Challenges and opportunities. IET Optoelectron..

[19] Hasan M.K., Chowdhury M.Z., Shahjalal M., Nguyen V.T., Jang Y.M. (2018). Performance Analysis and Improvement of Optical Camera Communication. Appl. Sci..

[20] Liu W., Xu Z. (2020). Some practical constraints and solutions for optical camera communication. Phil. Trans. R. Soc. A.

[21] Nguyen H., Nguyen V., Nguyen C., Bui V., Jang Y. (2021). Design and Implementation of 2D MIMO-Based Optical Camera Communication Using a Light-Emitting Diode Array for Long-Range Monitoring System. Sensors.

[22] Jurado-Verdu C., Guerra V., Matus V., Almeida C., Rabadan J. (2021). Optical Camera Communication as an Enabling Technology for Microalgae Cultivation. Sensors.

[23] Rátosi M., Simon G. (2020). Robust VLC Beacon Identification for Indoor Camera-Based Localization Systems. Sensors.

[24] Simon G., Zachár G., Vakulya G. (2017). Lookup: Robust and Accurate Indoor Localization Using Visible Light Communication. IEEE Trans. Instrum. Meas..

[25] Chavez-Burbano P., Guerra V., Rabadan J., Perez-Jimenez R. (2019). Optical Camera Communication system for three-dimensional indoor localization. Optik.

[26] (1897). Method of Measuring the Speed of Camera Shutters. Sci. Am..

[27] Kelley J.D. (1939). Camera Shutter Tester. U.S. Patent.

[28] Springer B.R. (1978). Camera Testing Methods and Apparatus. U.S. Patent.

[29] Fuller A.B. (1934). Electronic Chronometer. U.S. Patent.

[30] The Photoplug Optical Shutter Speed Tester for Your Smartphone. https://www.filmomat.eu/photoplug.

[31] ALVANDI Shutter Speed Tester. https://www.mr-alvandi.com/technique/Alvandi-shutter-speed-tester.html.

[32] Shutter Tester—7FR-80D. https://www.jpu.or.jp/eng/shutter-tester/.

[33] ISO 516:2019 Camera Shutters—Timing—General Definition and Mechanical Shutter Measurements. International Organization for Standardization, 2019. https://www.iso.org/obp/ui/#iso:std:iso:516:ed-4:v1:en.

[34] Asakura Y., Takahashi S., Doi K., Watanabe A., Ushiyama T., Inoue A. (1999). Exposure Precision Tester and Exposure Precision Testing Method for Camera. U.S. Patent.

[35] LaRue R.S. (1949). Shutter Speed Measurement Techniques. Master’s Thesis.

[36] Davidhazy A. Calibrating Your Shutters with TV Set and Turntable. https://people.rit.edu/andpph/text-calibrating-shutters.html.

[37] Budilov V.N., Volovach V.I., Shakurskiy M.V., Eliseeva S.V. Automated measurement of digital video cameras exposure time. Proceedings of the East-West Design & Test Symposium (EWDTS 2013).

[38] Masson L., Cao F., Viard C., Guichard F. Device and algorithms for camera timing evaluation. Proceedings of the IS&T/SPIE Electronic Imaging Symposium.

[39] Image Engineering LED-Panel. https://www.image-engineering.de/products/equipment/measurement-devices/900-led-panel.

[40] D’Antona G., Ferrero A. (2006). Digital Signal Processing for Measurement Systems: Theory and Applications.

[41] Shize G., Shenghe S., Zhongting Z. A novel equivalent sampling method using in the digital storage oscilloscopes. Proceedings of the 1994 IEEE Instrumentation and Measurement Technology Conference.

[42] Rátosi M., Vakulya G., Simon G. Measuring Camera Exposure Time Using Equivalent Sampling. Proceedings of the 2021 IEEE International Instrumentation and Measurement Technology Conference (I2MTC).

